# Comparison of Growth Rate and Nutrient Content of Five Microalgae Species Cultivated in Greenhouses

**DOI:** 10.3390/plants8080279

**Published:** 2019-08-10

**Authors:** Maria N. Metsoviti, George Papapolymerou, Ioannis T. Karapanagiotidis, Nikolaos Katsoulas

**Affiliations:** 1 Laboratory of Agricultural Constructions and Environmental Control, Department of Agriculture Crop Production and Rural Environment, University of Thessaly, Fytokou Street, 38446 Volos, Greece; 2 General Department, University of Thessaly, 41110 Larissa, Greece; 3 Aquaculture Laboratory, Department of Ichthyology and Aquatic Environment, University of Thessaly, Fytokou Street, 38446 Volos, Greece

**Keywords:** seasonal growth, biomass production, nutrient content, growth chamber, light intensity, temperature, *Chlorella vulgaris*

## Abstract

The effect of different environmental conditions on the growth rate, biomass production, nutrient composition, and phenolic content of the microalgae species *Chlorella vulgaris*, *Botryococcus braunii*, *Chlamydomonas reinhardtii*, *Euglena gracilis*, and *Nannochloropsis oculata* was investigated. The experiments were conducted in open bioreactors in a greenhouse in three different periods (during October, March, and June), and in a controlled environment in a closed plant growth chamber. It was found that the growth rate and production of *C. vulgaris* and *B. braunii* was higher during March, *C. reinhardtii* and *N. oculata* grew better in June, and the growth of *E. gracilis* was similar in March and June. The lipid content of the biomass of all five species increased with increasing light intensity and temperature, while the nitrogen free extractable (NFE) content decreased and the protein, fiber, moisture, and ash content remained relatively unaffected. The phenolic content varied from species to species with *E. gracilis* having the highest and *N. oculata* the lowest content among the species studied. The results can be taken into account when cultivating the different microalgae studied in full scale applications, such as in open raceway bioreactors, where conditions could be adjusted to obtain the most favorable growth conditions, depending on the particular species cultivated.

## 1. Introduction

Microalgae are unicellular photosynthetic organisms that use light and carbon dioxide, with a higher photosynthetic efficiency than plants, for the production of biomass. Their biodiversity is large; there are species that prosper in fresh water, such as *Chlorella vulgaris*, *Botryococcus braunii*, *Chlamydomonas reinhardtii*, and *Euglena gracilis*, and others in salt water, such as the marine microalgae species *Nannochloropsis oculata*. They are used in the pharmaceutical industry, in biodiesel production, in wastewater management, as nutritional supplements for human nutrition, and as feed for animals and fish [1]. They contain a high percentage of proteins (with a good amino acid profile) and lipids (with significant amounts of DHA (Docosahexaenoic Acid) and EPA (Eicosapentaenoic acid) that are essential for fish feeds), varying by algal species [2]. In addition, they have a high content of vitamins (such as A, B1, B2, B6, B12, C, E, biotin, folic acid) [3], inorganic salts (phosphate, zinc, iron, calcium, selenium, magnesium), antioxidants, and pigments such as chlorophylls, carotenoids, and phenolic compounds [4].

Phenolic compounds are a diverse class of secondary metabolites that contain a polyphenol structure consisting of two or more six-carbon aromatic rings [5]. They are considered as one of the most important classes of natural antioxidants because of their ability to donate a hydrogen atom or an electron in order to form stable radical intermediates [6]. The total phenolic content varies in different microalgae species and is influenced by environmental variations [7]. They play an important role in animal and human nutrition, and for this reason, their measurement in all species studied is important.

The microalgae culture systems are influenced by different factors, such as temperature, light intensity, carbon dioxide, pH, and the nutrient composition of the culture medium [8]. Among the environmental factors, light and temperature are two of the most important affecting algal growth and biomass production [9], with the optimal temperature and the requirements in light varying among the different algal species. When cultivated in stress conditions, such as inadequate nutrients in the culture medium, and/or very high or low light intensities and temperatures [10], a decrease in biomass production and in growth rate is observed [11].

The macronutrient composition, namely lipids, proteins, and carbohydrates of the microalgae biomass produced, is affected by the environmental and cultural condition variations, such as temperature, light intensity, pH, and nutrient composition of the culture medium [12,13]. In some microalgae species, the increase of light intensity resulted in a higher lipid content and decreased protein and carbohydrate content—such as in *B. braunii*, as reported by Ruangsomboon (2012) [14]—while in other species, low light conditions resulted in a high lipid content, such as in *Nannochloropsis* sp. [15] and *Ankistrodesmus falcatus* [16]. As far as the effect of temperature is concerned, in *Isochrysis galbana* [17] and *Scenedesmus obliquus* [18], lipid content increased with increasing temperature, while in *Chlorella vulgaris*, lipid content increased with decreasing temperature [19]. In the Australian species *Chaetoceros* sp., *Rhodomonas* sp., and *Cryptomonas* sp., the protein content decreased when cultivated at high temperatures, while a consistent trend was not observed in terms of carbohydrate content [20].

The present study was conducted in order to investigate the effect of different seasonal environmental conditions on five different microalgae species and to compare their relative growth rates and biomass productions, as well as their nutrient composition and the variation in total phenolic content. Therefore, five microalgae species, namely *Chlorella vulgaris*, *Botryococcus braunii*, *Chlamydomonas reinhardtii*, *Euglena gracilis*, and the marine species *Nannochloropsis oculata*, were cultivated in three different environmental conditions in a greenhouse, where temperature and solar irradiation were allowed to fluctuate naturally and additionally in a controlled environment-closed plant growth chamber, where the temperature was fixed and irradiation was artificial (with high pressure sodium lamps). These microalgae species were selected for study because, according to the literature, they are promising for fast growth and ease of adaptation to Greek environmental conditions. Additionally, they can potentially be used as animal or fish feeds due to their high lipid and protein content. To the best of our knowledge, data comparing the seasonal effect of cultivation on growth rate, nutrient content, and total phenolic content of the biomass of these five algal species are lacking. Rather than examining only one parameter of growth separately (temperature or light intensity), the seasonal effect reflects growth conditions encountered in nature, which combine both temperature and light intensity, assimilating natural conditions from very favorable to least favorable, encountered in nature by algae species.

## 2. Materials and Methods

### 2.1. Microalgae Cultivation

The microalgae species (SAG Strains Number: *C. vulgaris*: 211-11b, *B. braunii*: 30.81, *C. reinhardtii*: 11-32a, *E. gracilis*: 1224-5/25, *N. oculata*: 38.85) were obtained from the Experimental Phycology and Culture Collection of Algae from the University of Goettingen in Germany (EPSAG, Goettingen, Germany). They were cultivated in open bioreactors of 50 L capacity each, at three different environmental conditions in a greenhouse, where temperature and irradiation were allowed to fluctuate naturally. The bioreactors used in the experiments were rectangular Teflon vessels of 25 × 40 × 50 cm. Air, in the form of bubbles, was introduced into the cultivation medium through perforated tubing that was placed along the bottom of each bioreactor. The air was supplied during the entire cultivation period in each bioreactor at a rate of 200 L h^−1^, which corresponds to 0.08 L of CO_2_ h^−1^. An internal circulator was used for stirring the water.

The first experiment was conducted from October to November with an average temperature T_average_ = 23.2 °C, and average solar radiation Ι_average_ = 6.6 MJ m^−2^day^−1^. The second experiment was conducted from March to April with T_average_ = 28.5 °C, Ι_average_ = 15.9 MJ m^−2^day^−1^, and the third experiment was from June to July with T_average_ = 36.1 °C, Ι_average_ = 25.7 MJ m^−2^day^−1^. Additionally, cultivation was practiced in a controlled environment-closed plant growth chamber, where the temperature and light intensity were fixed (artificial irradiation with high pressure sodium lamps, MASTER GreenPower 600 W EL 400 V Mogul 1 SL, Philips, (Amsterdam, The Netherlands), Ι_average_ = 8.1 MJ m^−2^day^−1^, temperature set point of T = 24 °C). The light intensity and temperature conditions observed in the growth chamber were similar to those of the first experiment conducted in the greenhouse during autumn. Nevertheless, the spectrum of the high-pressure sodium lamps differed in several quality aspects with that of sunlight. High-pressure sodium lamps are rather poor in the blue wavelength compared to sunlight but provide substantial light in the green and yellow, as well as in the orange (600–620 nm). They also provide a substantial amount of red light (620–700 nm), although not as high as that provided by sunlight. Microalgae mostly contain chlorophylls a and b, which absorb strongly between 400–500 nm and between 600–680 nm. Therefore, the most substantial difference between the two types of illumination is in the quantity of blue light, which is higher in the Sun’s spectrum.

The greenhouse and growth chamber were set aside for these cultivation experiments only. No other experiments were conducted at the same place to minimize disturbance and avoid any possibility of contamination. The greenhouse was covered with glass. The air temperature and solar radiation inside the greenhouse were measured and recorded using a meteorological station (Pessl Instruments, Weiz, Austria) and the daily average values were calculated. Attention was paid so that all the bioreactors were exposed to similar lighting conditions. Three replications (open bioreactors) per treatment were performed.

In each experiment the culture medium was inoculated with a standard quantity (250 mL of microalgae inoculum), which was prepared as follows: 2 L Erlenmeyer flasks, containing the necessary culture medium, were inoculated with each microalgae culture directly obtained from EPSAG and cultivated in a sterile environment until they reached an absorbance reading of 0.5. The cultivation of the inoculum was always done under the same conditions, namely at a temperature of 25 °C, under natural illumination, and by using an orbital shaker at 60 rpm in order to prevent the sticking of algae to the surfaces of the flask. The acclimation in all three replications, which were performed simultaneously in each season, was further ensured by the lag phase that was present in all greenhouse cultivations and that had duration of about 3 to 8 days.

The microalgae species studied were grown in Basal Medium (Sammlung von Algenkulturen der Universität Göttingen (SAG), Göttingen, Germany) (= ES “Erddekokt + Salze”) containing the following nutrients per L: 0.2 g KNO_3_, 0.02 g K_2_HPO_4_, and 0.02 g MgSO_4_.7H_2_O. Additionally, it contained 30 mL of soil extract per liter that was prepared as follows: In a 6 L flask, one third was filled with garden or leaf soil of medium humus content that did not contain fertilizers or plant-protective agents. De-ionized water was added until it stood 5 cm above the soil and was sterilized by heating in a steamer for one hour twice in a 24 h interval. The decanted extract was separated from particles using centrifugation and was poured into small containers of stock solution, each of a size appropriate to making a batch of media, autoclaved for 20 min at 121 °C, and stored in the refrigerator. The Basal Medium also contained the following micronutrients: 0.005 mg L^−1^ ZnSO_4_.7H_2_O, 0.01 mg L^−1^ MnSO_4_.4H_2_O, 0.05 mg L^−1^ H_3_BO_3_, 0.005 mg L^−1^ Co(NO_3_)_2_.6H_2_O, 0.005 mg L^−1^ Na_2_MoO_4_.2H_2_O, 0.000025 mg L^−1^ CuSO_4_.5H_2_O, 3.5 mg L^−1^ FeSO_4_.7H_2_O, 4 mg L^−1^ EDTA, (Ethylenediaminetetraacetic acid), and 905 mL L^−1^ de-ionized water.

The marine species *N. oculata* was grown in Brackish Water Medium (Sammlung von Algenkulturen der Universität Göttingen (SAG), Göttingen, Germany) (= 1/2 SWES) containing the following nutrients per liter: 0.2 g KNO_3_, 0.02 g K_2_HPO_4_, 0.02 g MgSO_4_.7H_2_O, 30 mL of soil extract, and the quantity of micronutrients stated above. In addition, 450 mL L^−1^ of de-ionized water were added and 455 mL L^−1^ of seawater that was prepared with the addition of synthetic salt in order to reach a water salinity of 35‰. (Sammlung von Algenkulturen der Universität Göttingen (SAG), Göttingen, Germany; Culture Collection of Algae, Abteilung Experimentelle Phykologie und Sammlung von Algenkulturen (EPSAG), Universität Göttingen, Göttingen, Deutschland; official web page: http://epsag.uni-goettingen.de, 2007).

### 2.2. Measurements

The microalgae concentration was determined daily using optical density measurements at 655 nm with the use of a spectroscopy UV/Vis instrument. The spectrophotometer used for the measurement of absorbance was a Cintra 101 Model-GBC (Dandenong, Australia). Three samples were collected daily from each culture and all measures were carried out in triplicate. Additionally, samples taken from all cultures for the determination of absorbance were randomly subjected at all stages of cultivation (exponential and stationary growth phase) to examination in the microscope for the determination of any contamination as a matter of routine analyses. No contamination by bacteria or other species of algae was found. Also, both macroscopically and under the microscope, the pigmentation of algae was vividly green.

At the end of each experimental period, the total production of each culture was measured (in g L^−1^) after harvest of the biomass by a process, which was aided by raising the pH of the culture medium, in order to cause the algae to flocculate [21,22]. The pH was raised by adding sodium hydroxide, a base, which induces more than 90% flocculation at pH 11, according to Safi et al. [23]. After sedimentation, the supernatant medium was removed, the condensates were collected, and the excess water on them was evaporated in an air circulation oven at 40 °C until dry. The biomass was stored at −20 °C, and prior to biochemical analysis, it was pulverized using a planetary ball mill at 180 rpm for 10 min (FRITSCH pulverisette, Idar-Oberstein, Germany).

The specific growth rate in the exponential growth phase (μ_exp_, which is the slope of the growth rate curve in the exponential phase) was calculated according to the relation:

μ_exp_ = ln(α_2_/α_1_)/(t_2_ − t_1_) (1)

where, α_1_ and α_2_ are the absorbance readings at the beginning and the end of exponential growth phase, at time 1 (t_1_) and 2 (t_2_), respectively.

### 2.3. Nutrient Composition Analyses

The nutrient composition of the samples was determined according to AOAC (Association of Official Analytical Chemists) methods [24]. Specifically, moisture content of the samples was determined by drying the samples in an oven at 105 °C until a constant weight was obtained. Total protein content was calculated using the Kjeldahl method with a conversion factor of 6.25. To measure the total lipid content, lipids were extracted from the samples with 1:1 chloroform:methanol using the method of Folch et al. [25]. According to Ryckebosch et al. [26], chloroform:methanol 1:1 was shown to be the best solvent mixture for the extraction of total lipids from microalgae. Ash content was determined using incineration at 600 °C. After extracting lipids with petroleum ether, crude fiber content in the biomass was measured by boiling the samples: (a) in 0.128 M H_2_SO_4_ solution for 30 min, washing the remaining solids twice in hot water, and drying it in an oven at 105 °C; and (b) boiling using the same procedure in 0.128 M KOH solution, and also washing twice and drying in 105 °C. The remaining solids were weighed and then incinerated at 600 °C for at least three hours until a constant weight was obtained to determine the ash content. The difference in weight was the biomass content in crude fiber. Total phenolic content of each microalgae species was determined using a Folin–Ciocalteu reagent according to the Singleton and Rossi method [27], using gallic acid as a standard.

Nitrogen free extractable (NFE): The nitrogen-free extractable represents the non-structural carbohydrates, primarily of readily available carbohydrates, and any solubilized hemicellulose and lignin. They were calculated using the following equation:

%NFE = 100 − (%CP + %TL + %Ash + %CF + %Moisture) (2)

where: CP = crude protein, TL = total lipids, and CF = crude fiber

### 2.4. Statistical Analysis

Comparison of means was performed by subjecting the data to one-way analysis of variance at a significance level of 0.05 using the IBM SPSS Statistics 24 (Armonk, NY, USA) statistical package. The significant differences between treatments were determined using Tukey’s multiple comparison test.

## 3. Results

**Figure 1:**
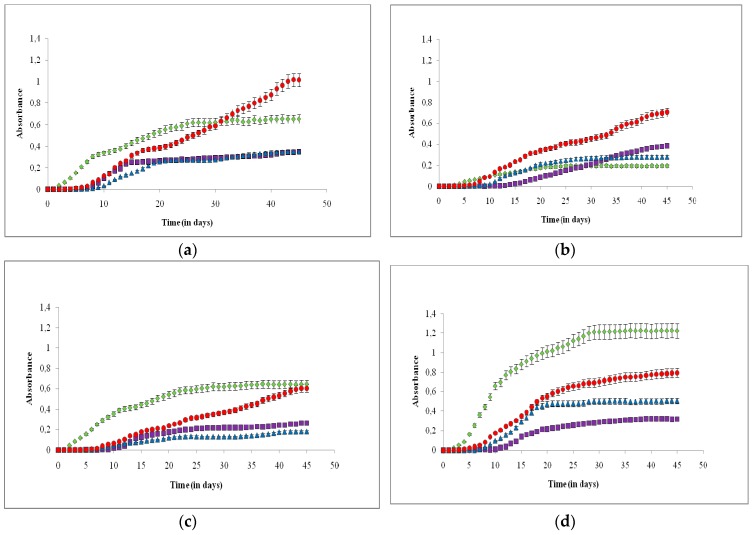

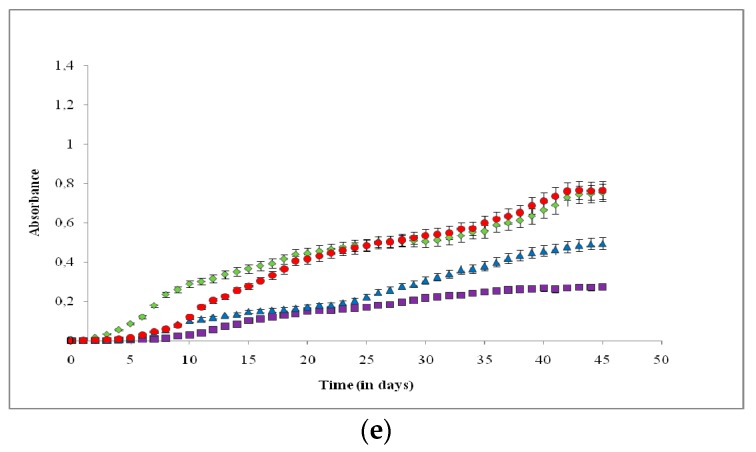
Growth curves of the five microalgae species: *C. vulgaris* (**a**), *B. braunii* (**b**), *C. reinhardtii* (**c**), *N. oculata* (**d**), and *E. gracilis* (**e**) in the four different environmental conditions. Red circles: March, Blue triangles: Controlled environment of growth chamber, Purple squares: October, Green diamonds: June. The error bars represent the standard deviation of the means.

Figure 1a–e illustrates the absorbance readings versus the cultivation time of each of the five species (*C. vulgaris*-a, *B. braunii*-b, *C. reinhardtii*-c, *N. oculata*-d, *E. gracilis*-e) during the cultivation in the four different environmental conditions (October, March, June, controlled environment). Table 1 presents the respective: (i) mean daily light intensity (in MJ/m^2^), (ii) mean temperature of the cultivation media (in °C), (iii) specific growth rates (μ_exp_) in exponential growth phases (in d^−1^), and (iv) biomass productions (P_b_ in g L^−1^), for the five microalgae species. The nutrient compositions of the biomass of the five species cultivated in the four different environmental conditions are presented in Table 2.

It was found that biomass growth rates and total productions varied for all five microalgae species in the different environments (Table 1). Cultivation in October and in the controlled environment of growth chamber did not favor fast growth rates in most of the five microalgae species. Cultivation of *C. vulgaris* was enhanced in March, while in June, the growth rate was higher than in October and the growth chamber, but not as high as it was in March. For *B. braunii*, the highest growth rate was found in March. The growth rate of *C. reinhardtii* in June was higher than it was in March. For *N. oculata*, the growth rate in June was substantially higher than it was in October and in the controlled environment of the growth chamber. In March, the growth rate was higher than in October and the growth chamber, but not as high as it was in June. For *E. gracilis*, cultivation in October and in the controlled environment of the growth chamber did not favor high growth rates, while cultivation during March and June resulted in higher growth rates and biomass production.

It was found (Table 2) that the light intensity and temperature did not substantially affect the protein, fiber, moisture, and ash content of the algal biomass. It should be mentioned that the nutrient composition of the culture medium was the same in all environmental conditions. On the other hand, there was an increase in the lipid content of the biomass of all five species with the increase of the mean daily light intensity and temperature (Table 1) while, the NFE content decreased.

Table 3 shows the variation in total phenolic content for the five microalgae species grown in the four different environmental conditions. It was found that seasonal variation in phenolic content varied from species to species. It was found that *E. gracilis*, on average, had the highest phenolic content and *C. vulgaris* had the second-highest phenolic content, while *N. oculata*, a salt water species, had the lowest phenolic content from the species studied.

## 4. Discussion

### 4.1. Effects on Growth Rate

Microalgae growth is affected by different factors [28], with temperature and light intensity being among the most important. Optimization of the conditions of cultivation can lead to production of adequate microalgae biomass [29]. In the present study, it was shown that the combination of light intensity and temperature had a profound effect not only on the growth rate and maximum absorbance, but also on the biomass production of all five species studied, with the results varying in each algal species according to the environmental conditions. Cultivation in October and in the controlled environment of growth chamber did not favor fast growth rates in most of the five microalgae species. In these two environmental conditions, the light intensities were low (Table 1) and this was probably a limiting factor for the growth of most species.

Cultivation of *C. vulgaris* was enhanced in March because the temperature and the light intensity were higher than in October and in the controlled environment of the growth chamber. In June, the growth rate was higher than in October and the growth chamber, but not as high as it was in March, because the temperatures and light intensities were very high. It is known that very high light intensity, as well as very high temperature, inhibits algal growth. For *C. vulgaris*, optimal growth occurs near 30 °C [30]. For *B. braunii*, the highest growth rate was found in March. In October and in the controlled environment of the growth chamber, the light intensity was not adequate for satisfactory biomass production in comparison with cultivation in the other two seasons. In June, the temperature was very high, and this was the limiting factor for the growth of *B. braunii*. For this algal species, the optimal temperature is near 25 °C [31]. The growth rate of *C. reinhardtii* in June was higher than it was in March. As in the previous species, the growth rates in October and in the controlled environment of the growth chamber were slow due to the lower temperatures and light intensities. It was found that *C. reinhardtii* grew better in higher temperatures and light intensities than the previous two algal species. For *N. oculata*, the growth rate in June was substantially higher than it was in October and in the controlled environment of the growth chamber. In March, the growth rate was higher than in October and the growth chamber, but not as high as it was in June. *N. oculata* grew better in higher temperatures and light intensities, like *C. reinhardtii*. For *E. gracilis*, cultivation in October and in the growth chamber did not favor fast growth rates because the mean temperature and irradiation were low. The increase in temperature and irradiation (cultivation during March and June) led to consequent increases in μ_exp_ and in biomass productions in comparison with the cultivation in October and in the growth chamber. These results are in agreement with Kitaya et al. [32], who reported that the optimum temperature for *E. gracilis* cultivation was in the range of 27–31 °C.

Temperature and light intensity are two important parameters that play a significant role in microalgae growth rate and production of all species [33]. Light contributes to cell multiplication, respiration, and photosynthesis [34]. Microalgae require light to produce ATP (Adenosine triphosphate) and NADPH (Nicotinamide adenine dinucleotide phosphate) and synthesize essential molecules for growth [35]. Biomass production in microalgae species generally increases with increasing light intensity, which is due to the higher absorption and utilization of photons by the photosynthetic apparatus. However, at high radiation intensities, beyond the saturation point, photo-inhibition is observed because of the photo-oxidation reactions taking place inside the cell [36]. This saturation point, as far as light intensity is concerned, depends on the particular algal species and the cultivation conditions. The effect of temperature on growth rate and biomass production is similar to the effect of light intensity, with the increase of temperature resulting in the increase of growth rate and production up to a certain level that varies from species to species [18].

### 4.2. Comparison of Macronutrient Composition

Lipids content of the algae species studied increased while the NFE content decreased with light and temperature increases, whereas protein, fiber, and ash content remained relatively unaffected. Light and temperature are among the most important environmental factors affecting the nutrient composition of the algal biomass, with the requirements varying among the different algal species. Temperature is an important factor that affects the synthesis of lipids [20,37]. Total lipid content in microalgae increases to a certain extent as the temperature increases and reaches an optimal level [38]. The optimal temperature (where the highest biomass and lipid production is achieved) varies from species to species, and as a result, it is difficult to generalize regarding the specific influence of the environmental factors on growth, biochemical composition, and enzyme activities in different microalgae species [39].

Lipid, and especially triacylglycerol, synthesis requires excess ATP and NADPH, which are produced by the photosynthesis process. When excess energy in the form of photons is supplied, more lipids can be synthesized, in which case they utilize this excess energy and therefore protect the algal cells from photochemical damage [40,41]. Therefore, when photon flux is increased, more carbon produced from the photosynthesis is used toward lipid production.

As far as the protein content of all microalgae species is concerned, it was found that the increase in light intensity and temperature did not follow the same trend as the lipid content, as protein content was similar (*p* < 0.05) in the four different environmental conditions for all five species. This may be due to protein being a significant structural and metabolic component of algal cells, such that their protein content might be more resistant to alterations of light intensity and temperature. Additionally, the protein content is affected more by nitrogen concentration, as nitrogen is required for protein synthesis and the nitrogen concentration of the culture medium was the same in all environmental conditions studied.

The effects of light intensity and temperature on nutrient composition vary among the different microalgae species. In some species, the increase of light intensity led to higher lipid content, such as for *Chlorella* sp. and *Monoraphidium* sp., as reported by He et al. [42], while in other species, low light intensities resulted in a high lipid content, such as for *Ankistrodesmus falcatus* [16]. Additionally, in *N. oculata* [19] and *S. obliquus* [18], the lipid content increased when cultivation was practiced in higher temperatures. Seyfabad et al. [43] reported that increased light intensity resulted in increased protein concentration in *C. vulgaris*, while Sharma et al. [44] reported that the protein content of *C. vulgaris* decreased when the microalgae was exposed to high light intensities. Similar findings with our study have been reported for the marine microalgae species *Odontella aurita*, in which cultivation in increased light intensity resulted in higher lipid content, but it had no significant effect on the protein content [45].

### 4.3. Comparison of Total Phenolic Content

The phenolic content of microalgae is affected by the cultivation conditions, such as temperature, light intensity, and availability of nutrients [46]. It should be mentioned that microalgae can produce significant amounts of phenols, which are substantially dependent on the particular species. In this study, the mean total phenolic content was found to vary substantially from species to species and was higher in *E. gracilis*, followed by *C. vulgaris*, *B. braunii*, *C. reinhardtii*, and lastly, *N. oculata*. Total phenolic content in *B. braunii* and *C. reinhardtii* were almost equal within experimental error. In the species *C. vulgaris*, *B. braunii*, and *C. reinhardtii*, the phenolic content was not highly dependent on the seasonal conditions. In *E. gracilis*, phenolic content was very low during growth in June. As such, it was found that high temperatures and light intensities did not favor production and accumulation of phenols in this microalgae species. The reverse was observed for *N. oculata*, where high light intensity and temperature substantially increased the phenolic content in the biomass. Goiris et al. [47] found that *C. vulgaris* contained from 800 to 2200 μg g^−1^ phenols and *N. oculata* and *B. braunii* contained about 2000 μg g^−1^ phenols. Cervantes-Garcia et al. [48] found that the biomass of *E. gracilis* contained about 137 μg g^−1^ phenols, while in our study, the phenolic content of almost up to 5800 μg g^−1^ was found in the biomass of *E. gracilis*. These differences in the phenolic content are probably due to different cultivation conditions and to the different strains studied. For comparison, the average content in phenols in red wine [49] and oregano [50] is 4000 μg g^−1^ and 2000 μg g^−1^, respectively.

## 5. Conclusions

Overall, the growth rate of the microalgae species studied was found to be highly dependent on seasonal environmental conditions. While, in most species growth is unfavorable during October, and differences in growth rate were found for March (spring) and June (summer): for *N. oculata* and *C. reinhardtii*, June growth conditions were more favorable, while for *B. braunii* and *C. vulgaris* March growth conditions were more favorable. In all algal species, it was found that the lipid content increased with the increase of light intensity and temperature, while the NFE content decreased, and the protein, fiber, and ash content remained relatively constant. The phenolic content was dependent both on the particular species as well as on the seasonal growth conditions. Among the species studied, total phenolic content in the *E. gracilis* biomass was higher compared to the other species, and that of *N. oculata* was had the lowest content. Thus, it was found that the natural environmental conditions significantly affected both the macronutrient and the phenolic content of microalgae biomass of the five species studied. The results of this study depended on the particular species grown as well as on the particular nutrient sought, which could be useful information for full scale applications, such as in open raceway bioreactors, where conditions could be adjusted to simulate the most favorable growth conditions.

## Figures and Tables

**Table 1:** Mean daily (and standard deviation) irradiation (I in MJ m^−2^ day^−1^), temperature of the cultivation media (T in °C), specific growth rates in exponential growth phase (μ_exp_ in d^−1^), and biomass productions (P_b_ in g L^−1^) for the five microalgae species cultivated in the four different environmental conditions.

Microalgae Species		October	Controlled Environment	March	June
	I	6.6 ± 2.8	8.1 ± 1.7	15.9 ± 5.3	25.7 ± 3.3
	T	23.2 ± 2.5	24.0 ± 1.1	28.5 ± 2.9	36.1 ± 3.2
*C. vulgaris*	μ_exp_	0.25 ± 0.01 ^c^	0.24 ± 0.01 ^c^	0.32 ± 0.01 ^a^	0.30 ± 0.01 ^b^
	P_b_	0.47 ± 0.01 ^c^	0.49 ± 0.00 ^c^	1.10 ± 0.01 ^a^	0.89 ± 0.01 ^b^
*B. braunii*	μ_exp_	0.19 ± 0.01 ^bc^	0.22 ± 0.01 ^b^	0.24 ± 0.00 ^a^	0.17 ± 0.02 ^c^
	P_b_	0.54 ± 0.00 ^b^	0.40 ± 0.01 ^c^	0.95 ± 0.00 ^a^	0.30 ± 0.01 ^d^
*C. reinhardtii*	μ_exp_	0.16 ± 0.01 ^c^	0.14 ± 0.01 ^d^	0.20 ± 0.01 ^b^	0.29 ± 0.01 ^a^
	P_b_	0.33 ± 0.01 ^c^	0.26 ± 0.01 ^d^	0.80 ± 0.00 ^b^	0.86 ± 0.01 ^a^
*N. oculata*	μ_exp_	0.15 ± 0.01 ^d^	0.20 ± 0.01 ^c^	0.23 ± 0.01 ^b^	0.31 ± 0.01 ^a^
	P_b_	0.43 ± 0.01 ^d^	0.66 ± 0.00 ^c^	0.98 ± 0.00 ^b^	1.28 ± 0.01 ^a^
*E. gracilis*	μ_exp_	0.16 ± 0.01 ^c^	0.19 ± 0.01 ^b^	0.25 ± 0.01 ^a^	0.26 ± 0.01 ^a^
	P_b_	0.37 ± 0.01 ^c^	0.69 ± 0.01 ^b^	0.96 ± 0.01 ^a^	0.95 ± 0.01 ^a^

Values represent averages ± std. deviation (n = 6). Values in the same row bearing different superscripts indicate a statistically significant difference (*p* < 0.05).

**Table 2:** Nutrient content (%) of the five microalgae species cultivated in the four different environmental conditions.

Microalgae Species	Nutrient Content %	October	Controlled Environment	March	June
*C. vulgaris*	Moisture	8.1 ± 0.2 ^a/A^	8.3 ± 0.1 ^a/AB^	8.2 ± 0.1 ^a/AB^	8.1 ± 0.2 ^a/A^
	Lipids	8.2 ± 0.4 ^a/AB^	9.3 ± 0.3 ^b/A^	15.0 ± 0.4 ^c/A^	20.3 ± 0.3 ^d/A^
	Proteins	25.1 ± 0.2 ^a/A^	25.4 ± 0.3 ^a/A^	25.5 ± 0.4 ^a/A^	25.9 ± 0.2 ^a/A^
	Ash	12.2 ± 0.3 ^a/AB^	12.7 ± 0.3 ^a/A^	12.1 ± 0.2 ^a/A^	12.3 ± 0.2 ^a/AB^
	Fiber	8.9 ± 0.3 ^a/A^	8.5 ± 0.1 ^ab/A^	8.2 ± 0.2 ^b/A^	8.1 ± 0.4 ^b/A^
	NFE	37.5 ± 0.5 ^a/A^	35.8 ± 0.3 ^b/A^	31.0 ± 0.1 ^c/A^	25.3 ± 0.3 ^d/A^
*B. braunii*	Moisture	8.4 ± 0.2 ^a/A^	8.6 ± 0.1 ^a/AB^	8.2 ± 0.1 ^a/AB^	8.3 ± 0.2 ^a/A^
	Lipids	7.4 ± 0.4 ^a/A^	8.6 ± 0.3 ^b/AB^	11.6 ± 0.2 ^c/B^	16.6 ± 0.5 ^d/B^
	Proteins	23.7 ± 0.3 ^a/BC^	23.8 ± 0.3 ^a/BC^	23.7 ± 0.1 ^a/B^	23.4 ± 0.1 ^a/B^
	Ash	10.9 ± 0.4 ^a/C^	10.9 ± 0.1 ^a/B^	10.1 ± 0.2 ^b/B^	10.4 ± 0.1 ^ab/C^
	Fiber	11.5 ± 0.2 ^a/CD^	11.4 ± 0.2 ^a/B^	11.1 ± 0.4 ^a/B^	10.9 ± 0.2 ^a/BC^
	NFE	38.1 ± 0.4 ^a/A^	36.7 ± 0.2 ^b/A^	35.3 ± 0.2 ^c/B^	30.4 ± 0.1 ^d/B^
*C. reinhardtii*	Moisture	8.3 ± 0.2 ^a/A^	8.8 ± 0.1 ^b/B^	8.2 ± 0.2 ^a/AB^	8.2 ± 0.2 ^a/A^
	Lipids	8.3 ± 0.4 ^a/AB^	9.2 ± 0.5 ^a/AB^	13.8 ± 0.4 ^b/CD^	15.8 ± 0.4 ^c/B^
	Proteins	24.3 ± 0.4 ^a/AB^	24.0 ± 0.3 ^a/B^	24.3 ± 0.3 ^a/C^	24.5 ± 0.2 ^a/C^
	Ash	11.5 ± 0.4 ^ab/BC^	11.7 ± 0.2 ^b/C^	11.1 ± 0.1 ^a/C^	11.4 ± 0.1 ^ab/D^
	Fiber	12.1 ± 0.4 ^a/D^	11.8 ± 0.1 ^ab/B^	11.7 ± 0.3 ^ab/B^	11.5 ± 0.1 ^b/C^
	NFE	35.5 ± 0.2 ^a/B^	34.5 ± 0.6 ^a/B^	30.9 ± 0.6 ^b/A^	28.6 ± 0.1 ^c/C^
*N. oculata*	Moisture	8.2 ± 0.3 ^a/A^	8.1 ± 0.2 ^a/AB^	8.4 ± 0.2 ^a/A^	8.3 ± 0.2 ^a/A^
	Lipids	8.5 ± 0.2 ^a/B^	11.5 ± 0.4 ^b/C^	14.8 ± 0.5 ^c/AD^	18.8 ± 0.4 ^d/C^
	Proteins	19.3 ± 0.5 ^a/D^	19.4 ± 0.2 ^a/D^	19.6 ± 0.1 ^a/D^	19.7 ± 0.2 ^a/D^
	Ash	12.3 ± 0.3 ^a/AB^	12.5 ± 0.2 ^a/A^	12.8 ± 0.3 ^a/D^	12.6 ± 0.1 ^a/A^
	Fiber	10.3 ± 0.4 ^a/B^	10.1 ± 0.1 ^a/C^	10.2 ± 0.2 ^a/C^	10.4 ± 0.3 ^a/B^
	NFE	41.4 ± 0.5 ^a/C^	38.4 ± 0.3 ^b/C^	34.2 ± 0.1 ^c/C^	30.2 ± 0.2 ^d/B^
*E. gracilis*	Moisture	8.1 ± 0.2 ^a/A^	7.8 ± 0.1 ^a/A^	7.9 ± 0.1 ^aB^	7.8 ± 0.2 ^a/A^
	Lipids	7.4 ± 0.4 ^a/A^	8.3 ± 0.3 ^b/B^	13.1 ± 0.4 ^c/C^	17.8 ± 0.7 ^d/C^
	Proteins	23.0 ± 0.2 ^a/C^	23.2 ± 0.3 ^a/C^	23.9 ± 0.7 ^b/BC^	23.3 ± 0.1 ^a/B^
	Ash	12.7 ± 0.3 ^ab/A^	12.9 ± 0.2 ^b/A^	12.3 ± 0.3 ^ab/AD^	12.2 ± 0.2 ^a/B^
	Fiber	11.1 ± 0.3 ^a/BC^	11.4 ± 0.3 ^a/B^	11.2 ± 0.2 ^a/B^	10.8 ± 0.2 ^a/B^
	NFE	37.7 ± 0.4 ^a^	36.4 ± 0.5 ^b/A^	31.6 ± 0.3 ^c/A^	28.1 ± 0.8 ^d/D^

Values represent averages ± std. deviation (n = 6). Values in the same row (lowercase superscripts) or column (capital superscripts) bearing different superscripts indicate a statistically significant difference (*p* < 0.05). Lowercase superscripts are for comparison between the same species in the different environmental conditions and capital superscripts are for comparison between specific nutrients among the different species in the same environmental condition.

**Table 3:** Total phenolic content (TPC) (in μg GAE- Gallic acid equivalent) g^−1^ dry biomass) of the five microalgae species cultivated in the four different environmental conditions.

Microalgae Species	Total Phenolic Content (TPC) (μg GAE g^−1^ dry biomass)			
	October	Controlled Environment	March	June
*C. vulgaris*	3629 ± 21 ^a^	2703 ± 24 ^b^	3024 ± 31 ^c^	2469 ± 21 ^d^
*C. reinhardtii*	2406 ± 16 ^a^	2469 ± 20 ^b^	2124 ± 19 ^c^	2476 ± 12 ^b^
*B. braunii*	2342 ± 11 ^a^	2775 ± 13 ^b^	2144 ± 23 ^c^	2321 ± 13 ^a^
*N. oculata*	1318 ± 26 ^a^	1115 ± 14 ^b^	912 ± 14 ^c^	2906 ± 16 ^d^
*E. gracilis*	5806 ± 10 ^a^	5741 ± 18 ^b^	4166 ± 15 ^c^	2815 ± 18 ^d^

Values represent averages ± std. deviation (n = 6). Values in the same row bearing different superscripts indicate a statistically significant difference (*p* < 0.05).
